# Development and validation of a cefepime population pharmacokinetic model to guide treatment for patients with hospital-acquired pneumonia requiring renal replacement

**DOI:** 10.1128/aac.00900-26

**Published:** 2026-10-08

**Authors:** Adrian Valadez, Emma Harlan, Marc H. Scheetz, Michael N. Neely, Helen K. Donnelly, Richard G. Wunderink, Nathaniel J. Rhodes

**Affiliations:** 1 Department of Pharmacy Practice, Midwestern University, College of Pharmacy 15475, Downers Grove, Illinois, USA; 2 Pharmacometrics Center of Excellence, Midwestern University 3541, Downers Grove, Illinois, USA; 3 Department of Pharmacy, Northwestern Memorial Hospital 24560, Chicago, Illinois, USA; 4 Department of Pharmacology, College of Graduate Studies, Midwestern University 3541, Downers Grove, Illinois, USA; 5 Department of Biomedical Sciences, College of Graduate Studies, Midwestern University 69281, Downers Grove, Illinois, USA; 6 Keck School of Medicine, University of Southern California 5116, Los Angeles, California, USA; 7 Laboratory of Applied Pharmacokinetics and Bioinformatics, The Saban Research Institute, Children's Hospital Los Angeles 5150, Los Angeles, California, USA; 8 Division of Pulmonary and Critical Care Medicine, Department of Medicine, Northwestern University Feinberg School of Medicine 12244, Chicago, Illinois, USA; University of Houston, Houston, Texas, USA

**Keywords:** pharmacokinetics, pneumonia, Monte Carlo simulation, renal replacement

## Abstract

Cefepime is used in critically ill pneumonia patients, but dosing in renal replacement therapy (RRT) is complicated by altered renal function, extracorporeal removal, and variability in pulmonary epithelial lining fluid (ELF) exposure. We sought to develop an integrated model linking systemic, extracorporeal, and pulmonary cefepime pharmacokinetics in RRT. Pharmacokinetic (PK) data from critically ill adults receiving cefepime and requiring RRT were included. In a dedicated continuous RRT (CRRT) cohort (*n* = 8), pre- and post-filter plasma and effluent PK data were collected. Additional patients (*n* = 43) requiring RRT also contributed plasma and ELF samples. A non-parametric population PK model was developed using an 80/20 subject-level development-validation split. Covariate models assessed the impact of residual renal function, effluent flow rate, body size, and fixed intradialytic hemodialysis (HD) clearance. Monte Carlo simulations evaluated plasma and ELF target attainment across dosing strategies, weight strata, and minimum inhibitory concentrations (MICs). The final model jointly described systemic, extracorporeal, and pulmonary cefepime disposition using piecewise central volume, native clearance, linear effluent-flow-dependent CRRT clearance, fixed HD clearance, and ELF distribution. Simulations showed adequate plasma target attainment, whereas ELF attainment was sensitive to MIC, target, regimen, and body weight. Continuous infusions of 4 g/day provided adequate ELF attainment at MICs ≤4 mg/L. Plasma-based cefepime target attainment may overestimate pulmonary exposure during RRT, particularly at higher MICs, body weights, and *f*T>MIC targets. External validation and prospective studies are needed to refine pulmonary target estimates and determine whether plasma-guided Bayesian dosing can reliably ensure site-of-infection exposure.

## INTRODUCTION

Cefepime is a hydrophilic, time-dependent β-lactam antibiotic with low protein binding and predominant renal elimination that is commonly used for severe gram-negative infections, including hospital-acquired and ventilator-associated pneumonia. (1) In critically ill patients, pharmacokinetics can be highly variable because of dynamically changing renal function, altered volume of distribution, fluid shifts, and extracorporeal support, including renal replacement therapy (RRT). (1) Continuous RRT (CRRT) further complicates dosing because extracorporeal clearance impacts total drug elimination, which may impact target-site concentrations. Additionally, drug dosing in CRRT is frequently extrapolated from plasma-based pharmacokinetic (PK)/pharmacodynamic (PD) targets (2–6), which may overestimate target attainment.

Cefepime exposures in patients requiring RRT are influenced by multiple factors, including residual clearance and extracorporeal factors (e.g., modality, blood flow, membrane properties, and effluent intensity) (7–9). Effluent intensity is typically weight based, such that absolute effluent flow rate and corresponding drug removal may differ substantially across body-weight strata (10, 11). Because cefepime is minimally protein bound (2) and readily cleared by extracorporeal therapy, CRRT-related determinants may meaningfully affect systemic exposure and, by extension, the probability of achieving PK/PD targets. However, the relative contribution of residual clearance, extracorporeal clearance, and distributional changes remains difficult to predict in practice. Prior research has evaluated cefepime plasma PK and target attainment in CRRT (7, 8, 10–13). However, plasma PK exposures may not mirror those within the epithelial lining fluid (ELF), which represents the pulmonary site of infection in pneumonia patients. Plasma and ELF exposures may diverge, meaning that an assumed (static) ELF-to-plasma penetration ratio can lead to inadequate dosing. (14) Knowledge of plasma (pre- and post-filter), CRRT effluent, and ELF PK data within an integrated framework could inform clinically actionable cefepime dosing recommendations for patients requiring RRT.

Herein, we developed and internally validated a cefepime population pharmacokinetic model integrating pre- and post-filter plasma, CRRT effluent, and ELF concentrations for application in patients requiring RRT. Among patients on CRRT, we simulated plasma and ELF target attainment across varying cefepime dosing strategies, weight-standardized effluent rates, and PK/PD targets.  

## MATERIALS AND METHODS

### Patients and data sources

The current study included data from patients enrolled in Successful Clinical Response in Pneumonia Therapy (SCRIPT), a prospective observational study conducted at a single academic medical center (NIH grant U19AI135964) (15), and patients enrolled in a prospective CRRT substudy of the parent RX-SCRIPT study (NIH grant R01AI158530 and FDA contract 75F40122C00134).

Adult ICU patients with suspected or confirmed pneumonia who were treated with cefepime at the discretion of their primary clinical team and contributed at least one plasma concentration were eligible for inclusion. Patients contributing observations while receiving CRRT were classified as undergoing CRRT for those observations. Patients requiring intermittent hemodialysis (HD) who were not undergoing CRRT at the time of sampling were classified as HD patients for those observations. Concurrent extracorporeal membrane oxygenation was not an exclusion criterion. Patients who received cefepime but did not require renal replacement therapy during the observed treatment course were excluded from analysis.

Pharmacokinetic data sources included (i) timed PK samples collected from patients enrolled in the prospective CRRT substudy, (ii) banked plasma and/or ELF samples from patients enrolled in SCRIPT, and (iii) salvaged residual clinical plasma specimens. In the prospective CRRT substudy, plasma samples were collected immediately before the next dose and at 0.5, 1, 2, 4, or 6 h after the start of infusion, with effluent samples collected concurrently when available. During the prospective study period, cefepime doses were administered over 0.5 h per institutional protocols. Nominal within-occasion sampling schedule among the richly sampled cohort was obtained within a single treatment course. Patients could contribute samples across multiple dosing occasions. Patients enrolled in SCRIPT contributed ELF samples to the analysis.

### Intermittent hemodialysis therapy

Intermittent HD was modeled using a fixed clearance in the primary analysis due to the sparse nature of available intradialytic samples (see “Population Pharmacokinetic Model”). This clearance was activated only during intradialytic periods. Interdialytic clearance was estimated as a random parameter in the population model shared across patients (i.e., systemic clearance). Observations were assigned to intradialytic and interdialytic periods based on documentation contained in clinical flowsheets within the EHR.

### Continuous renal replacement therapy

CRRT was delivered according to local clinical practice using the Prismaflex System platform (Baxter Healthcare, Deerfield, IL, USA) equipped with an HF1400 hemofilter (polyarylethersulfone membrane, surface area 1.4 m^2^, priming volume 165 mL). (16) The HF1400 filter has an *in vitro* ultrafiltration coefficient of 43 mL/h/mmHg and a maximum blood flow rate of 400 mL/min. Effluent intensity, dialysis, and replacement fluid rates were prescribed by the treating clinical team. Blood entering and exiting the extracorporeal circuit was sampled as pre-filter and post-filter plasma, respectively, and CRRT effluent samples were collected concurrently when available.

### Bioanalytical methods

Cefepime concentrations in pre- and post-filter plasma and CRRT effluent were quantified using a validated liquid chromatography-tandem mass spectrometry assay performed on an Agilent Ultivo triple quadrupole mass spectrometer. The effluent assay used the same chromatographic conditions, extraction procedures, and calibration range as the plasma assay. Chromatographic separation was achieved using a Kinetex 2.6 µm Polar C18 column (50 × 2.1 mm) maintained at 25°C. Mobile phases consisted of water with 0.1% formic acid and acetonitrile. Gradient elution was performed at a flow rate of 0.7 mL/min with a maximum operating pressure of 500 bar. The system was held at 98% aqueous phase for 2 min, transitioned to 95% organic phase and held for 1 min, and then returned to initial conditions for reequilibration through 4 min. Deuterated cefepime (cefepime-d3) was used as the internal standard. Mass transitions were monitored in positive electrospray ionization using selected reaction monitoring, with precursor/product ion pairs of *m*/*z* 242.6 → 88/87 for cefepime-d3 and *m*/*z* 241.1 → 85/84 for cefepime. The lower limit of quantification was 0.5 µg/mL. The assay was linear from 1 to 100 µg/mL (*R*^2^ = 0.999). Accuracy (interday: 99.2%–101.9%, intraday: 97.9%–102.8%) and precision (interday coefficient of variation [CV%]: 0.3%–1.7%, intraday CV%: 0.8%–1.1%) met FDA requirements for bioanalytical method validation. (17) Samples exceeding the upper limit of the calibration range were diluted and reanalyzed using a standard laboratory procedure. Assay error equations were used to model proportional error.

Cefepime BAL assays were performed by the clinical laboratory at Northwestern Memorial Hospital using a synthetic BAL matrix. Calibration curves were linear from 0.04 to 10 mg/L. Urea BAL assays were developed using ^15^N₂-urea as an internal standard, with calibration ranges of 0.2–50 mg/L. Assays were validated using three independent qualification runs with acceptance criteria of less than 15% coefficient of variation for accuracy and precision, except at the lower limit of quantification, where less than 20% was accepted. (17)

Plasma urea assays were completed using the Abnova kit according to manufacturer instructions. ELF concentrations were estimated from BAL concentrations using urea dilution correction as shown in equation 1.


(1) $$C_{ELF}=\;C_{BAL\;}\times \;\frac{{urea}_{plasma}}{{urea}_{BAL}}$$

where $C_{BAL}$ is the measured cefepime concentration in BAL fluid and ${urea}_{plasma}$ and ${urea}_{BAL}$ are the corresponding urea concentrations in plasma and BAL fluid.

### Data set processing

Dose histories, infusion durations, sample times, observed concentrations, and clinical covariates were assembled into a unified data set for population PK analysis. Observation records were coded by biological matrix as pre-filter plasma, post-filter plasma, CRRT effluent concentration, cumulative effluent amount, or ELF. Covariates carried forward into model development included body weight, renal function, renal replacement modality, CRRT effluent flow rate, and relevant extracorporeal support indicators. Within-patient time-varying covariates were aligned using the last observation carried forward (LOCF). CRRT and intermittent HD status were treated as interval-based treatment states rather than conventional covariates; after LOCF, remaining interval values were resolved using the next observation carried backward to preserve the recorded treatment-state intervals. Intermittent HD was coded as active only during recorded intradialytic periods. CRRT modality was summarized across available treatment records, and patients who changed modalities during follow-up were counted under each modality received.

The modeling data set was divided into a development cohort and a held-out validation cohort using an 80/20 split in R. The split was stratified by cohort membership to preserve representation of richly sampled CRRT observations and additional plasma/ELF observations across the development and validation data sets. The development cohort was used for structural model development and final parameter estimation. The held-out validation cohort was used for internal validation by applying the final model without reestimating the population support-point locations.

### Population pharmacokinetic model

A compartment population PK model was developed in Pmetrics 3.0.9, with parameter estimation performed using the non-parametric adaptive grid algorithm (18, 19). The structural model (Fig. S1) included central and peripheral plasma compartments, the ELF compartment, and state variables describing CRRT effluent concentrations and cumulative cefepime removal in effluent. A piecewise central volume structure was evaluated a priori because patients could contribute observations during both on-circuit and off-circuit periods. This structure allowed central volume to differ according to time-varying CRRT status rather than forcing a single central volume parameter across pharmacologically distinct support states. Central volume was modeled as shown in equation 2:


(2) $$V_{1}=\;\left[V_{off}\times (1-CRRT)\right]+\;\left[V_{on}\times \;CRRT\right]$$

where $V_{off}$ represents the central volume term when the patient was not receiving CRRT; $V_{on}$ represents the central volume during CRRT; and CRRT is a time-varying indicator variable.

Residual non-CRRT clearance was modeled as shown in equation 3:


(3) $${CL}_{resid}=\;{CL}_{1}$$

where ${CL}_{1}$ represents residual systemic clearance not attributed to HD or CRRT clearance. CRRT-associated clearance was modeled as a linear function of the recorded effluent flow rate, which was converted from milliliter per hour to liter per hour, and scaled as shown in equations 4 and 5


(4) $$Q_{effluent}=\left(\frac{flow}{1,000}\right)\;\times \;CRRT$$

(5) $${CL}_{CRRT}=\;Q_{effluent}\;\times \;S_{eff}$$

where *Q*_effluent_ represents time-varying CRRT effluent flow during CRRT-active periods, and $S_{eff}$ is the estimated CRRT effluent clearance scalar, which is functionally analogous to an apparent sieving or saturation coefficient that scales effluent flow to CRRT-associated clearance. Because it was estimated rather than directly calculated, it is referred to as an effluent-clearance scalar.

Intradialytic HD clearance ${CL}_{HD}$ was fixed at 7.2 L/h during HD events as a literature value. (20) Due to the sparse nature of PK samples around HD events, intradialytic HD clearance was treated as a fixed value in the primary analysis. Sensitivity analyses evaluated values of 3.6 and 10.8 L/h.

Total cefepime clearance from the central compartment was modeled as shown in equation 6


(6) $${CL}_{T}=\;{CL}_{resid}+\;{CL}_{HD}+{CL}_{CRRT}$$

where ${CL}_{T}$ is the linear and state-dependent combination of ${CL}_{resid}$, ${CL}_{HD}$, and ${CL}_{CRRT}$ estimates. Post-filter plasma concentrations were modeled by scaling pre-filter plasma concentrations by a factor during CRRT-active periods as shown in equation 7:


(7) $$C_{post}=C_{pre}\times \;S_{post}\;\times CRRT$$

where $S_{post}$ is the estimated post-filter scalar. A $S_{post}$ < 1 indicates the fraction of pre-filter concentrations returning to systemic circulation after traversal of the hemofilter circuit. The overall elimination rate from the central compartment was estimated as shown in equation 8


(8) $$K_{e}=\;\frac{{CL}_{T}}{V_{1}}$$

thereby linking all observed concentrations within a single model framework. Residual unexplained variability was described using proportional error polynomials for each output, with final error model weighting estimated using the multiplicative error term (*γ*).

### Model development and covariate evaluation

A pre-specified piecewise central volume structure was selected based on our knowledge of CRRT physiology and the available longitudinal on- and off-circuit sampling. Candidate covariate structures were evaluated to determine whether conventional renal-function estimates or body-size descriptors improved model performance. The focused development candidate set included (i) the piecewise central volume model with systemic residual clearance, (ii) a model with fixed linear creatinine-clearance scaling on systemic residual clearance, (iii) a model with body-weight scaling on the non-CRRT central volume term only, and (iv) a model with body-weight scaling on systemic residual clearance only. The focused covariate evaluation was designed to test whether body size and renal function improved model performance. Specifically, body-weight effects were tested separately for off-circuit central volume and systemic residual clearance to reduce the risk that weight-related volume or clearance effects would be confounded by extracorporeal factors.

Model comparisons were performed using −2 log-likelihood, Akaike information criterion (AIC), diagnostic performance, parameter plausibility, and intended simulation purpose. Because the focused candidate models had the same number of parameters, AIC differences paralleled differences in −2 log-likelihood and were interpreted as relative support among candidate structures rather than as formal proof of covariate effects. Final development model selection was based on minimization of AIC, goodness-of-fit diagnostics for outputs, and the rule of parsimony.

### Model evaluation and Bayesian forecasting

Model performance in the development cohort was evaluated using observed-vs-posterior individual-predicted diagnostic plots stratified by biological matrix. Predictive performance was assessed separately for pre-filter plasma, post-filter plasma, and ELF using coefficients of determination, regression intercepts, and regression slopes.

For internal validation, the final development model was applied to the held-out validation cohort as the prior population distribution. Subject-specific posterior parameter probabilities were estimated conditional on observed validation data without reestimating the population-support point locations, which evaluated the extent to which the final model support points could serve as an informative Bayesian prior. Predictive performance was summarized using the same metrics as the development model.

### Monte Carlo simulations

Monte Carlo simulations were performed using the final development model. Semiparametric sampling from the weighted non-parametric joint parameter distribution was used to generate 1,000 plasma and ELF concentration-time profiles per dosing strategy and body-weight stratum (21). Simulated profiles were evaluated over the 24- to 48-h post-treatment interval using model predictions generated from 23.9 to 48 h at 0.1 h intervals. Effluent flow rates were scaled to discrete total body-weight strata to maintain 30 mL/kg/h (corresponding to 1.8, 2.4, and 3.6 L/h) for simulated patients weighing 60, 80, and 120 kg. Three dosing strategies were evaluated: cefepime 2 g every 12 h as an intermittent infusion over 0.5 h with loading dose, cefepime 2 g every 12 h as an extended infusion (EI) over 4 h with loading dose, and cefepime 4 g every 24 h over 24 h as a continuous infusion with loading dose. In all simulations, an LD was given over 0.5 h.

Probability of target attainment (PTA) was defined as the proportion of simulated concentration-time profiles achieving the pre-specified percentage of free time above the minimum inhibitory concentration (MIC). Pharmacodynamic targets of 50%, 68%, and 100% *f*T>MIC were evaluated with 20% protein binding assumed; (2) target attainment was evaluated for both plasma and ELF using a free fraction of 0.8 (i.e., 1-protein binding). A benchmark of 90% probability of target attainment was used. Variability of MIC- and target-specific attainment rates was summarized using 95% confidence intervals.

### Use of AI tools

Generative artificial intelligence tools were used to assist with language editing and code review. All scientific content, analyses, interpretations, and final text were reviewed and approved by the authors.

## RESULTS

### Study population and sample distribution

The pooled data set comprised 51 critically ill adults and was partitioned into a development cohort of 40 patients and a held-out validation cohort of 11 patients. The final data set included pre-filter plasma, post-filter plasma, CRRT effluent concentration, cumulative effluent amount, and urea-corrected ELF observations collected across the dosing interval. Baseline characteristics are summarized in Table 1. The cohort was medically complex, with a median age of 67 years, median weight of 80.5 kg, median serum creatinine of 2.2 mg/dL, and median creatinine clearance of 39.2 mL/min. Most patients were male (62.7%). Renal replacement therapy was common, with 40 subjects (78.4%) undergoing CRRT and 16 (31.4%) undergoing hemodialysis at some point during the observed treatment course; CVVHDF was the predominant CRRT modality. Mutually exclusive RRT groups included 35 CRRT-only, 11 HD-only, and 5 patients who received both modalities; corresponding development counts were 27, 8, and 5, and validation counts were 8, 3, and 0, respectively.

**TABLE 1:** Baseline and clinical characteristics^*c*^*^,^*^*d*^

Demographics^*a*^	Overall (*n* = 51)	Development (*n* = 40)	Validation (*n* = 11)
Age (years)	67.0 (55.5–74.5)	67.0 (55.8–74.2)	64.0 (55.5–74.5)
Height (cm)	167.6 (158.3–177.8)	167.6 (157.9–177.8)	170.2 (161.3–176.6)
Weight (kg)	80.5 (70.0–97.7)	81.5 (73.1–97.6)	72.5 (63.1–92.0)
SCr (mg/dL)	2.2 (1.1–3.0)	2.0 (1.0–2.9)	2.6 (2.4–3.5)
BSA (m^2^)	1.9 (1.8–2.1)	1.9 (1.9–2.1)	1.9 (1.7–2.1)
CrCl (mL/min)	39.2 (27.0–73.0)	47.3 (28.6–85.2)	30.2 (21.6–36.8)
Flow (L/h) at initial CRRT	2.8 (2.2–3.4)	3.0 (2.3–3.5)	2.6 (2.2–2.9)
ECMO, *n* (%)	1 (2.0)	1 (2.5)	0 (0.0)
Ever underwent HD, *n* (%)	16 (31.4)	13 (32.5)	3 (27.3)
Ever underwent CRRT, *n* (%)^*b*^	40 (78.4)	32 (80.0)	8 (72.7)
CVVH, *n* (%)	2 (3.9)	2 (5.0%)	0 (0.0%)
CVVHD, *n* (%)	1 (2.0)	1 (2.5)	0 (0.0)
CVVHDF, *n* (%)	38 (74.5)	30 (75.0)	8 (72.7)
Sex, *n* (%)			
Male	32 (62.7)	24 (60.0)	8 (72.7)
Female	19 (37.3)	16 (40.0)	3 (27.3)

^*a*^ Continuous variables are presented as median (interquartile range), and categorical values as *n *(%).

^*b*^ CRRT modality categories are not mutually exclusive; one patient underwent more than one CRRT modality during follow-up.

^*c*^ SCr, serum creatinine; BSA, body surface area; CrCl, creatinine clearance; ECMO, extracorporeal membrane oxygenation; CVVH, continuous venous hemofiltration; CVVHD, continuous venovenous hemodialysis; CVVHDF, continuous venovenous hemodiafiltration.

^*d*^ Renal replacement therapy categories are not mutually exclusive. Patients were counted as having undergone CRRT or HD if they received the modality at any point during the observed treatment course; therefore, some patients contributed to both categories.

Sampling by cohort and matrix is summarized in Table S1. Pre-filter plasma observations were available across the prospective (*n* = 8) and opportunistically sampled (*n* = 43) cohorts, whereas post-filter plasma and effluent observations were from the prospective cohort. ELF observations were contributed primarily by the opportunistic cohort. Median PK observation follow-up was longer in the prospective cohort than in the opportunistic cohort in both the development and validation splits: 355.3 vs 160.2 h in development and 189.2 vs 63.5 h in validation. Details of the CRRT intensity, effluent flow rates, and CRRT duration are included in Table S2. The median initial CRRT effluent flow rate was 2.8 L/h. Time-averaged CRRT intensity was similar in the development and validation cohorts: 31.2 (IQR 29.2–35.0) vs 31.5 (IQR 29.9–33.6) mL/kg/h.

### Development model

The five-compartment structural model (Fig. S1), parameterized with piecewise central volume and residual clearance, served as the base model. Alternative covariate parameterization models were weaker by AIC and lacked objective or mechanistic justification to supplant the base model (Table S3), which was therefore retained as the final model. Sensitivity analyses of varying fixed HD clearance values did not improve model performance vs a fixed value of 7.2 L/h (data not shown).

Median parameter estimates (95% CrI) and CV% are summarized in Table 2, with the full support-point distribution shown in Table S4. The median (CV%) central volume was lower on-circuit vs off-circuit (7.44 L vs 8.69 L, CV 62% and 70%, respectively). The median estimated ELF scaling volume was 31.46 L (CV 56%). The median residual clearance estimate was 1.38 L/h (CV 32%). Sensitivity analyses excluding HD-only patients did not alter the median residual clearance estimate (data not shown). The median estimates for the effluent and post-filter plasma scalars were 0.82 (CV 17.5%) and 0.85 (21.4%), respectively.

**TABLE 2:** Median parameter values and 95% credible intervals from the final population PK model^*c*^

Parameter^*a*^	Median	Non-parametric CV%	95% CrI
*V*_on_ (L)	7.44	62	7.44–10.52
*V*_off_ (L)	8.69	70	8.69–9.96
*V*_ELF_ (L)	31.46	56	26.73–44.34
CL_resid_ (L/h)	1.38	32	1.23–1.38
CL_HD_ (L/h)	7.2	Fixed	Fixed
CL_CRRT_ (L/h)*^b^*	2.3	NA	Derived
*K*_12_ (h^−1^)	4.75	41	4.75–5.8
*K*_21_ (h^−1^)	7.57	34	6.95–7.57
*K*_15_ (h^−1^)	3.41	56	3.41–6.81
*K*_51_ (h^−1^)	5.25	62	1.77–5.38
*S*_eff_	0.82	17	0.71–0.87
*S*_post_	0.85	21	0.76–0.85

^*a*^ Median and 95% CrI were calculated from the weighted final population support point distribution. Non-parametric CV% summarizes the dispersion of the final population parameter distribution.

^*b*^ Calculated CL_CRRT_ derived from the median *S*_eff_ and median effluent flow rate of 2.8 L/h.

^*c*^ *V*_on_ represents the CRRT-on central volume term; *V*_off _represents the CRRT-off central volume term; *V*_ELF_ represents the ELF scaling volume; CL_resid_ represents residual systemic clearance; *S*_eff_ represents the CRRT effluent clearance scalar; *S*_post_ represents the post-filter plasma scalar; CrI, credible interval.

### Model predictive performance

**Fig 1:**
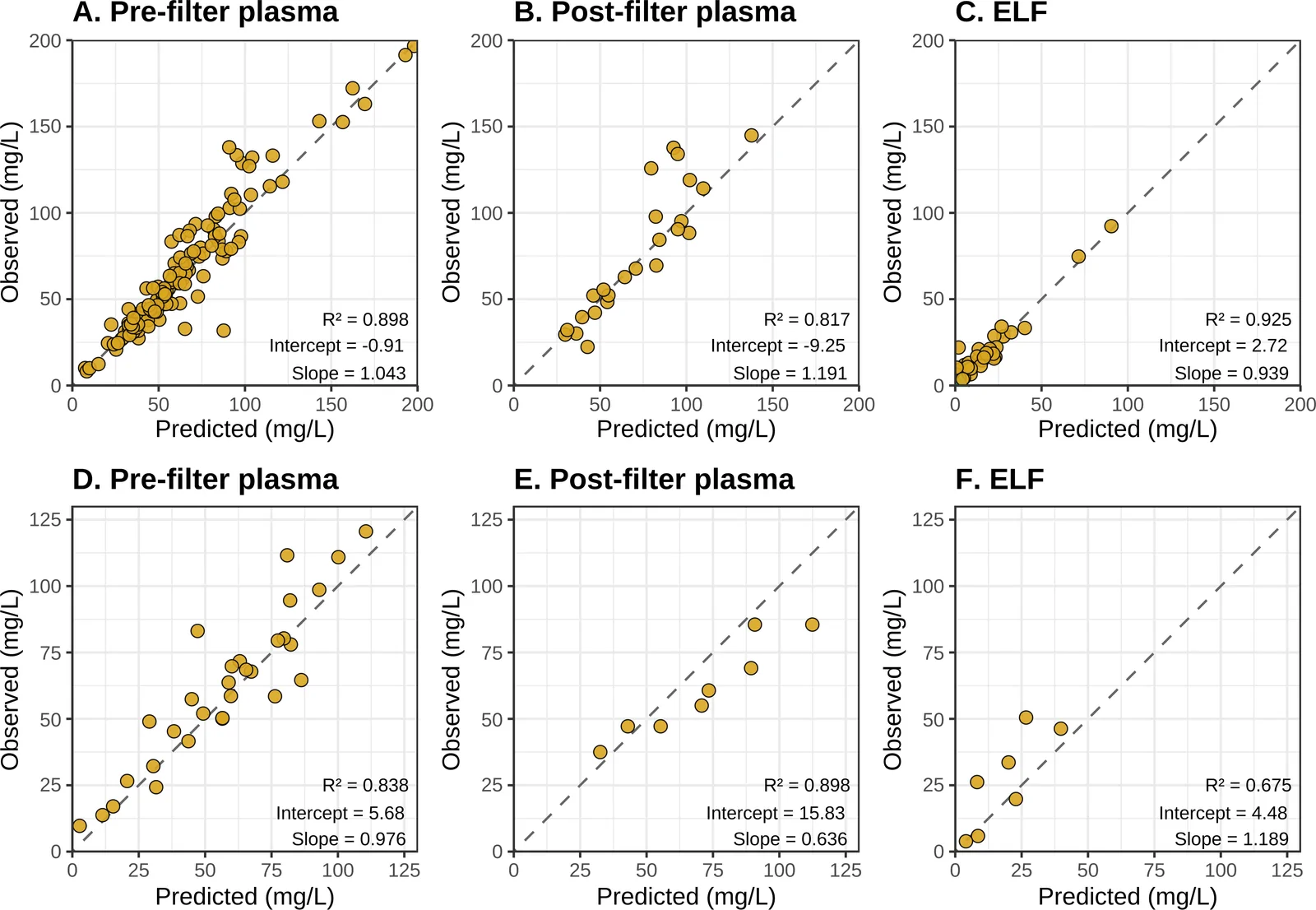
Observed vs posterior individual-predicted cefepime concentrations in the development and validation cohorts across biological matrices. Observed cefepime concentrations are plotted against posterior individual-predicted concentrations for pre-filter plasma, post-filter plasma, and ELF. (**A–C**) Development cohort and (**D–F**) held-out validation cohort. (**A and D**) Pre-filter plasma, (**B and E**) post-filter plasma, and (**C and F**) ELF. Matrix-specific coefficients of determination, intercepts, and slopes are displayed within each panel. *R*^2^, coefficient of determination.

Observed-vs-posterior individual-predicted concentrations in the development and validation cohorts are shown in Fig. 1. In the development and validation cohorts, diagnostics generally demonstrated acceptable agreement for plasma matrices, with slightly greater deviation from the line of unity among post-filter predictions in the held-out cohort. The performance of the model in predicting ELF concentrations was modest in the held-out cohort (Fig. 1F). No trends were observed in the individual weighted residual plots by time or concentration in the validation cohort (Fig. S2).

### Simulated plasma and ELF target attainment across dosing strategies

**Fig 2:**
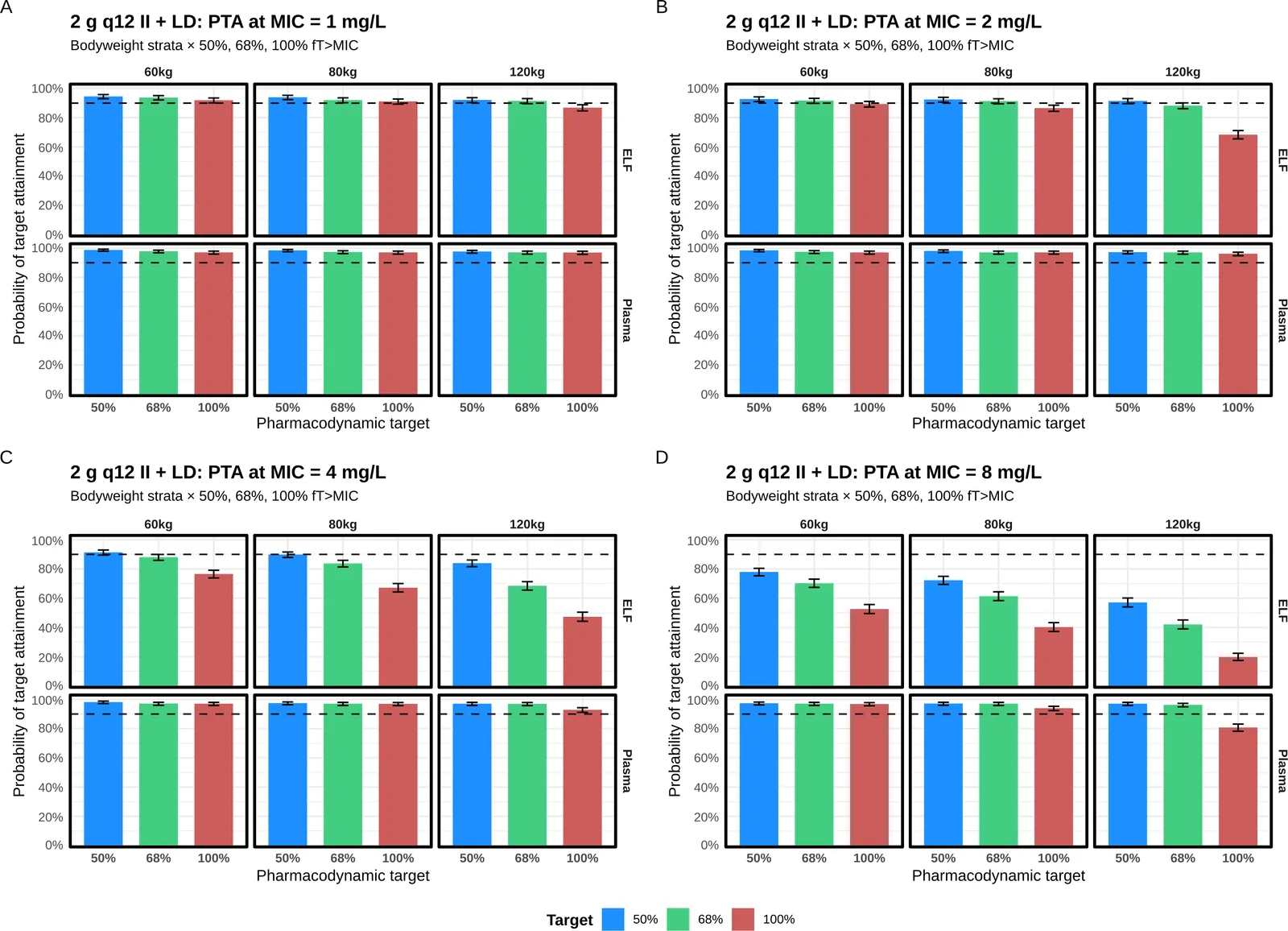
Probability of target attainment for cefepime 2 g q12h intermittent infusion with loading dose. PTA for cefepime 2 g every 12 h as an intermittent infusion with a loading dose across MIC values of 1, 2, 4, and 8 mg/L. (**A–D**) Increasing MIC values. PTA is shown by body-weight stratum, pharmacodynamic target, and sampling matrix. Rows compare ELF and plasma target attainment. Error bars represent simulation uncertainty, and the dashed horizontal line denotes 90% PTA. Clinical susceptibility breakpoints for cefepime vary by organism and interpretive standard; therefore, no single susceptibility breakpoint is shown for these organism-independent PTA simulations. II, intermittent infusion; LD, loading dose

**Fig 3:**
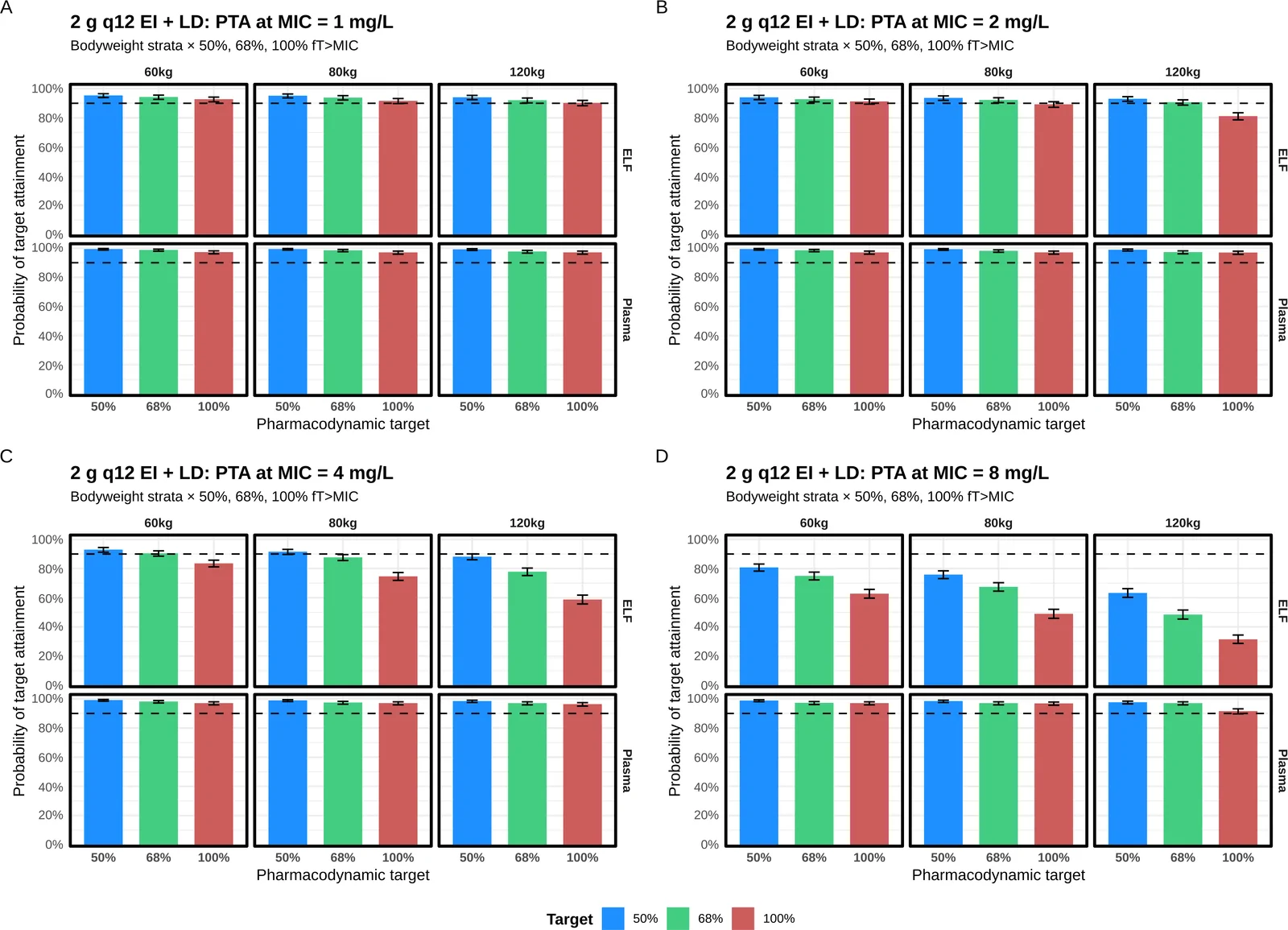
Probability of target attainment for cefepime 2 g q12h extended infusion with loading dose. PTA for cefepime 2 g every 12 h as an extended infusion with loading dose across MIC values of 1, 2, 4, and 8 mg/L. (**A–D**) Increasing MIC values. PTA is shown by body-weight stratum, pharmacodynamic target, and sampling matrix. Rows compare ELF and plasma target attainment. Error bars represent simulation uncertainty, and the dashed horizontal line denotes 90% PTA. Clinical susceptibility breakpoints for cefepime vary by organism and interpretive standard; therefore, no single susceptibility breakpoint is shown for these organism-independent PTA simulations. LD, loading dose.

**Fig 4:**
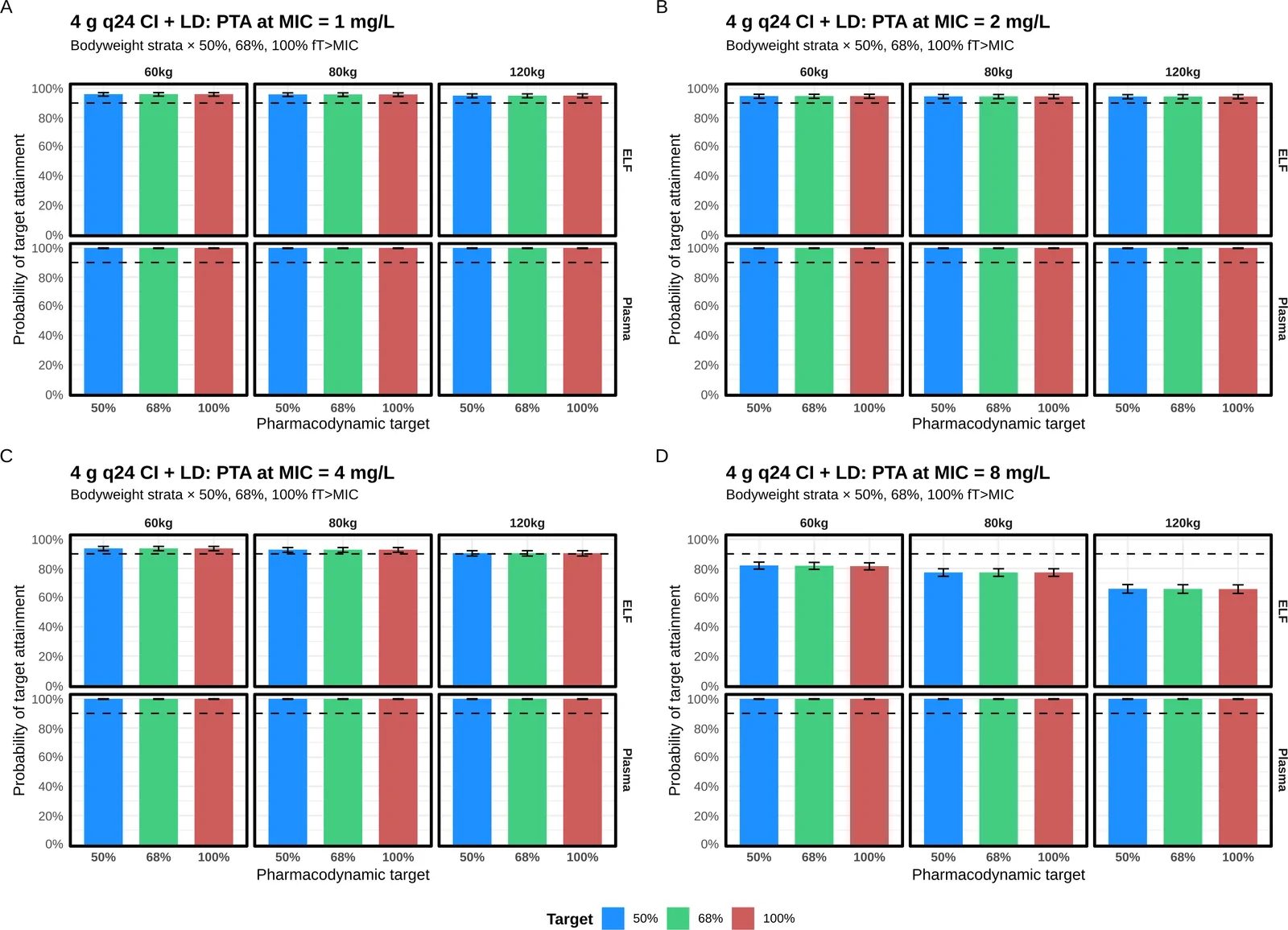
Probability of target attainment for cefepime 4 g 24 h continuous infusion with loading dose. PTA for cefepime 4 g over 24 h as a continuous infusion with a loading dose across MIC values of 1, 2, 4, and 8 mg/L. (**A–D**) Increasing MIC values. PTA is shown by body-weight stratum, pharmacodynamic target, and sampling matrix. Rows compare ELF and plasma target attainment. Error bars represent simulation uncertainty, and the dashed horizontal line denotes 90% PTA. Clinical susceptibility breakpoints for cefepime vary by organism and interpretive standard; therefore, no single susceptibility breakpoint is shown for these organism-independent PTA simulations. CI, continuous infusion; LD, loading dose.

Regimen-specific PTA at MIC values of 1, 2, 4, and 8 mg/L is shown for intermittent infusion, extended infusion, and continuous infusion in Fig. 2 to 4, respectively. Target attainment was frequently acceptable in plasma up to an MIC of 8 mg/L, with the exception of intermittent infusion dosing in the 120 kg patient (100% *f*T>MIC target). On the other hand, ELF target attainment was regimen-, target-, and body weight dependent as MICs increased. Results of the individual regimen simulations are detailed below.

For cefepime 2 g every 12 h as an intermittent infusion with loading dose, ELF PTA was acceptable at MICs of 1 and 2 mg/L for less stringent PK/PD targets but fell below 90% at MICs of 4–8 mg/L. At MICs of 8 mg/L, ELF PTA was consistently lower than plasma PTA, with the greatest reductions observed in the 120 kg patient and the 100% *f*T>MIC target. For cefepime 2 g every 12 h as an extended infusion with loading dose, ELF PTA fell below 90% at an MIC of 8 mg/L for all simulated body weights and PK/PD targets. For cefepime 4 g every 24 h as a continuous infusion with loading dose, ELF PTA was consistently >90% at an MIC ≤4 mg/L; however, at MICs of 8 mg/L, ELF PTA fell below 90%, whereas plasma PTA remained above 90% across body weights and PK/PD targets. Assessing PTA across MICs from 0.25 to 32 mg/L (Fig. S3 to S5), we found that plasma PTA remained adequate at MICs up to 8 mg/L in general, whereas ELF PTA was frequently below 90% at MICs of 4–8 mg/L, with the highest PTA observed for 4 g/24 h continuous infusion dosing.

## DISCUSSION

We developed and internally validated an RRT-specific cefepime population pharmacokinetic model that incorporated central and peripheral plasma compartments, an ELF compartment, CRRT effluent compartments, an a priori piecewise central volume structure, native clearance, and post-filter and effluent scaling terms. Our model is unique in that we were able to link systemic exposure, extracorporeal removal, and pulmonary exposure in a single population framework. Goodness-of-fit diagnostics generally supported that our plasma model was an informative Bayesian prior for held-out patients; however, ELF predictions carried greater uncertainty than plasma predictions. Our simulations revealed that plasma attainment did not uniformly predict ELF target attainment, particularly as MIC increased and targets became more stringent. Continuous infusion produced the most stable ELF exposure profile among the evaluated strategies, suggesting that prolonged maintenance of cefepime concentrations may partially mitigate, but not eliminate, plasma-ELF target attainment discordance.

Our model structure attempted to capture relevant physiology in patients on RRT. The piecewise central volume was selected to accommodate the time-varying structure of the data (i.e., patients were sampled both on and off circuit). Alternative structures such as shared central volume were considered but did not improve model fit (data not shown). Another reason for the selection of the piecewise volume structure is related to potential confounding arising from the fact that volume overload is a frequent indication for CRRT in unstable patients. Thus, Vd could differ with fluid status and illness severity. The potential for collider bias arises because CRRT effluent prescriptions are frequently weight-indexed but are also influenced by clinical factors related to CRRT initiation and intensity. By separating on-circuit and off-circuit disposition, we were able to test whether body weight or renal function added explanatory value in the off-circuit state. Although weight scaling of Vd was not ultimately supported in our model, this does not imply that weight was biologically unimportant, since information about weight is carried by the prescribed effluent rate.

The estimated CRRT scaling factors were internally consistent with measurable transmembrane cefepime removal. The median CRRT effluent clearance scalar of 0.82 indicated that CRRT-associated cefepime clearance was approximately 82% of recorded effluent flow rather than a one-to-one reflection of nominal effluent flow. At the cohort median initial effluent flow of 2.8 L/h, this corresponded to an estimated CRRT cefepime clearance of approximately 2.3 L/h. The median post-filter plasma scalar of 0.85 indicates that post-filter plasma concentrations were approximately 85% of corresponding pre-filter central plasma concentrations during CRRT-active periods. Thus, the model estimated both measurable cefepime recovery in effluent and a corresponding post-filter concentration decrement, supporting directional agreement between the effluent and post-filter observations and remaining broadly consistent with the relatively low protein binding of cefepime (e.g., 15%–20%). (2) Our model did not explicitly account for filter age, membrane fouling, delivered vs prescribed flow, or dynamic changes in circuit performance. However, the model retained reasonable predictive performance for pre- and post-filter plasma concentrations, with greater uncertainty for ELF, in the validation cohort (Fig. S2).

Human pulmonary PK studies support caution when extrapolating from plasma to ELF exposure. Boselli et al. reported similar mean steady-state plasma and ELF cefepime concentrations during continuous infusion in severe nosocomial pneumonia, suggesting high pulmonary penetration under those conditions, averaging 100%. (22) By contrast, Rodvold et al. reported a lower ELF-to-total plasma AUC ratio for cefepime in healthy adults receiving cefepime-zidebactam, underscoring that pulmonary exposure estimates can depend on population, administered regimen, and sampling design (23). Importantly, Rohani et al. found that plasma target attainment can overestimate pulmonary attainment in β-lactam-treated patients (14). Thus, the assumption of a fixed pulmonary penetration ratio, while clinically convenient, may be misleading in acutely ill patients.

Our study results are generally aligned with previous studies of cefepime in RRT but provide new insights into pulmonary target attainment (7, 8). Philpott et al. prospectively demonstrated that cefepime 2 g every 8 h as a 4-h extended infusion in critically ill adults undergoing CRRT achieved 100% *f*T>MIC at an MIC of 8 mg/L in plasma. (12) Al-Shaer and colleagues then built on those observations using population-PK modeling and simulations, which showed that aggressive extended-infusion regimens were most reliable for achieving systemic cefepime targets across a range of CRRT flow rates (10, 13). Our simulations suggest that plasma exposures may be adequate with high-dose EI regimens, yet pulmonary exposures may be suboptimal, particularly as MIC increases, suggesting that plasma attainment may be misleading for organisms with higher MICs.

Several limitations of our study should be acknowledged. First, although the model incorporated multiple biological matrices, the number of patients contributing ELF data was smaller than the number contributing plasma observations. ELF simulation results should be interpreted as exploratory and hypothesis generating, whereas the plasma target attainment findings are more strongly supported by the held-out validation. In addition, validation was internal and therefore does not establish generalizability to patient populations, institutions, CRRT practices, or treatment conditions not represented in the present study. Likewise, the limited number of richly sampled CRRT participants introduces uncertainty regarding the stability and generalizability of the estimated CRRT clearance relationship across broader CRRT populations and practices. Second, urea diffusion during bronchoalveolar lavage can introduce bias into ELF estimates, which are also sensitive to lavage technique, dwell time, and sample handling. (24) Third, a fixed estimate of 0.8 for the free fraction of cefepime was used for simulations. Although this is reasonable, given cefepime’s generally low protein binding, protein binding may vary in critically ill patients, which could alter realized free-drug target attainment (1, 25). Fourth, the continuous-infusion simulations were pharmacokinetic and did not incorporate formulation-specific stability constraints. Fifth, the ELF scaling-volume distribution was broad; therefore, this parameter should not be interpreted as a literal anatomical ELF volume. Rather, it should be interpreted as a model-estimated scaling parameter. The number of paired ELF and plasma observations was insufficient to support a reliable fixed pulmonary penetration ratio or regression relationship in our data set; therefore, we consider the model-based target attainment analyses a more appropriate translation of ELF exposure for clinical interpretation. The broad distribution of ELF-related parameters reflects uncertainty inherent to sparse pulmonary sampling and heterogeneous pulmonary exposure in critically ill patients.

### Conclusion

In this RRT-specific cefepime population pharmacokinetic analysis, a combined population PK model integrated knowledge of dialysis modality, pre-filter plasma, post-filter plasma, effluent, and ELF concentrations to predict systemic and pulmonary target attainment. We found that plasma target attainment was not equivalent to pulmonary target attainment at higher MICs and in patients with higher body weights receiving a CRRT intensity of 30 mL/kg/h. Internal validation metrics supported our model’s potential as a Bayesian prior for model-informed precision dosing, although external validation is needed. Future studies that integrate prospective paired plasma and ELF sampling are required to determine whether plasma-guided Bayesian dosing can reliably protect site-of-infection exposure in pneumonia patients requiring RRT.

## Data Availability

The clinical data underlying this study were collected per protocol (Northwestern University IRB STU00204868). Due to the sensitive nature of the patient data and institutional policies, data sets are not publicly available. De-identified data may be accessed by qualified researchers subject to a formal Data Use Agreement. Please contact a member of the SCRIPT study team (https://script.northwestern.edu/contact-us/) and the corresponding author to coordinate data access. To support transparency and reproducibility, the data analysis code has been made publicly available at https://github.com/njrhodes/FDA_FEP_public/.

## SUPPLEMENTAL MATERIAL

Supplemental material aac.00900-26-s0001.pdf Tables S1 to S4; Fig. S1 to S5.

ASM does not own the copyrights to Supplemental Material that may be linked to, or accessed through, an article. The authors have granted ASM a non-exclusive, world-wide license to publish the Supplemental Material files. Please contact the corresponding author directly for reuse.

## References

[1] Pais GM, Chang J, Barreto EF, Stitt G, Downes KJ, Alshaer MH, Lesnicki E, Panchal V, Bruzzone M, Bumanglag AV, Burke SN, Scheetz MH. 2022. Clinical pharmacokinetics and pharmacodynamics of cefepime. Clin Pharmacokinet 61:929–953. doi:10.1007/s40262-022-01137-y PMC9345683 35764774

[2] Anonymous. 2012. MAXIPIME (cefepime hydrochloride, USP) for injection [prescribing information]. Hospira, Inc. Lake Forest. IL. USA

[3] Craig WA. 1995. Interrelationship between pharmacokinetics and pharmacodynamics in determining dosage regimens for broad-spectrum cephalosporins. Diagn Microbiol Infect Dis 22:89–96. doi:10.1016/0732-8893(95)00053-d 7587056

[4] Maguigan KL, Al-Shaer MH, Peloquin CA. 2021. Beta-lactams dosing in critically Ill patients with gram-negative bacterial infections: A PK/PD approach. Antibiotics (Basel) 10:1154. doi:10.3390/antibiotics10101154 PMC8532626 34680734

[5] Nicasio AM, Ariano RE, Zelenitsky SA, Kim A, Crandon JL, Kuti JL, Nicolau DP. 2009. Population pharmacokinetics of high-dose, prolonged-infusion cefepime in adult critically ill patients with ventilator-associated pneumonia. Antimicrob Agents Chemother 53:1476–1481. doi:10.1128/AAC.01141-08 PMC2663089 19188394

[6] Pai Mangalore R, Peel TN, Udy AA, Peleg AY. 2023. The clinical application of beta-lactam antibiotic therapeutic drug monitoring in the critical care setting. J Antimicrob Chemother 78:2395–2405. doi:10.1093/jac/dkad223 PMC10566322 37466209

[7] Allaouchiche B, Breilh D, Jaumain H, Gaillard B, Renard S, Saux MC. 1997. Pharmacokinetics of cefepime during continuous venovenous hemodiafiltration. Antimicrob Agents Chemother 41:2424–2427. doi:10.1128/AAC.41.11.2424 PMC164139 9371344

[8] Malone RS, Fish DN, Abraham E, Teitelbaum I. 2001. Pharmacokinetics of cefepime during continuous renal replacement therapy in critically ill patients. Antimicrob Agents Chemother 45:3148–3155. doi:10.1128/AAC.45.11.3148-3155.2001 PMC90796 11600370

[9] Schetz M, Ferdinande P, Van den Berghe G, Verwaest C, Lauwers P. 1995. Pharmacokinetics of continuous renal replacement therapy. Intensive Care Med 21:612–620. doi:10.1007/BF01700172 7593908

[10] Al-Shaer MH, Maguigan K, Ashton J, Venugopalan V, Droege ME, Philpott CD, Droege CA, Healy DP, Mueller EW, Peloquin CA. 2022. Applying cefepime population pharmacokinetics to critically Ill patients receiving continuous renal replacement therapy. Antimicrob Agents Chemother 66:e01611-21. doi:10.1128/AAC.01611-21 PMC8765233 34662194

[11] Carlier M, Taccone FS, Beumier M, Seyler L, Cotton F, Jacobs F, Roberts JA. 2015. Population pharmacokinetics and dosing simulations of cefepime in septic shock patients receiving continuous renal replacement therapy. Int J Antimicrob Agents 46:413–419. doi:10.1016/j.ijantimicag.2015.05.020 26208469

[12] Philpott CD, Droege CA, Droege ME, Healy DP, Courter JD, Ernst NE, Harger NJ, Foertsch MJ, Winter JB, Carter KE, Van Fleet SL, Athota K, Mueller EW. 2019. Pharmacokinetics and pharmacodynamics of extended-infusion cefepime in critically Ill patients receiving continuous renal replacement therapy: a prospective, open-label study. Pharmacotherapy 39:1066–1076. doi:10.1002/phar.2332 31549737

[13] Al-Shaer MH, Philpott CD, Droege CA, Courter JD, Healy DP, Droege ME, Ernst NE, Mueller EW, Peloquin CA. 2021. Cefepime population pharmacokinetics and target attainment in critically Ill patients on continuous renal replacement therapy. Antimicrob Agents Chemother 65:e00144-21. doi:10.1128/AAC.00144-21 PMC8316051 33722885

[14] Rohani R, Yarnold PR, Scheetz MH, Neely MN, Kang M, Donnelly HK, Dedicatoria K, Nozick SH, Medernach RL, Hauser AR, Ozer EA, Diaz E, Misharin AV, Wunderink RG, Rhodes NJ. 2023. Individual meropenem epithelial lining fluid and plasma PK/PD target attainment. Antimicrob Agents Chemother 67:e00727-23. doi:10.1128/aac.00727-23 PMC10720524 37975660

[15] SCRIPT Investigators. 2026. Successful clinical response in pneumonia therapy (SCRIPT). Northwestern University. Available from: https://script.northwestern.edu/database

[16] Baxter Healthcare Corporation. 2015. PRISMAFLEX system HF1000/HF1400 hemofilter sets [product specification sheet]. Deerfield. IL. USA

[17] Department of Health and Human Services FaDA. 2018. Center for drug evaluation and research (CDER), Center for veterinary medicine (CVM). Bioanalytical method validation guidance for industry

[18] Neely MN, van Guilder MG, Yamada WM, Schumitzky A, Jelliffe RW. 2012. Accurate detection of outliers and subpopulations with Pmetrics, a nonparametric and parametric pharmacometric modeling and simulation package for R. Ther Drug Monit 34:467–476. doi:10.1097/FTD.0b013e31825c4ba6 PMC3394880 22722776

[19] Yamada WM, Neely MN, Bartroff J, Bayard DS, Burke JV, Guilder M van, Jelliffe RW, Kryshchenko A, Leary R, Tatarinova T, Schumitzky A. 2020. An algorithm for nonparametric estimation of a multivariate mixing distribution with applications to population pharmacokinetics. Pharmaceutics 13:42. doi:10.3390/pharmaceutics13010042 PMC7823953 33396749

[20] Maynor LM, Carl DE, Matzke GR, Gehr TWB, Farthing C, Farthing D, Brophy DF. 2008. An *in vivo*-*in vitro* study of cefepime and cefazolin dialytic clearance during high-flux hemodialysis. Pharmacotherapy 28:977–983. doi:10.1592/phco.28.8.977 18657014

[21] Goutelle S, Bourguignon L, Maire PH, Van Guilder M, Conte JE Jr, Jelliffe RW. 2009. Population modeling and Monte Carlo simulation study of the pharmacokinetics and antituberculosis pharmacodynamics of rifampin in lungs. Antimicrob Agents Chemother 53:2974–2981. doi:10.1128/AAC.01520-08 PMC2704682 19380594

[22] Boselli E, Breilh D, Duflo F, Saux MC, Debon R, Chassard D, Allaouchiche B. 2003. Steady-state plasma and intrapulmonary concentrations of cefepime administered in continuous infusion in critically ill patients with severe nosocomial pneumonia. Crit Care Med 31:2102–2106. doi:10.1097/01.CCM.0000069734.38738.C8 12973166

[23] Rodvold KA, Gotfried MH, Chugh R, Gupta M, Patel A, Chavan R, Yeole R, Friedland HD, Bhatia A. 2018. Plasma and intrapulmonary concentrations of cefepime and zidebactam following intravenous administration of WCK 5222 to healthy adult subjects. Antimicrob Agents Chemother 62:e00682-18. doi:10.1128/AAC.00682-18 PMC6105785 29784852

[24] Marcy TW, Merrill WW, Rankin JA, Reynolds HY. 1987. Limitations of using urea to quantify epithelial lining fluid recovered by bronchoalveolar lavage. Am Rev Respir Dis 135:1276–1280. doi:10.1164/arrd.1987.135.6.1276 3592404

[25] Al-Shaer MH, Alghamdi WA, Graham E, Peloquin CA. 2020. Meropenem, cefepime, and piperacillin protein binding in patient samples. Ther Drug Monit 42:129–132. doi:10.1097/FTD.0000000000000675 31318843

