# Supplementary material for: Development and validation of a cefepime population pharmacokinetic model to guide treatment for patients with hospital-acquired pneumonia requiring renal replacement
Source: Antimicrob Agents Chemother. 2026 Oct 8;70(11):e00900-26. doi: 10.1128/aac.00900-26 (PMC13731174; doi:10.1128/aac.00900-26)
Supplement: Supplementary file 1 [file aac.00900-26-s0001.pdf]

Supplemental Material

Table S1. Samples by Cohort and Matrix

	Samples (n)	***Subjects (n)***
Prospective sampling		
ELF	**1**	**1**
Effluent Concentration	**16**	**6**
Effluent mg (volume x conc)	**16**	**6**
Pre-filter	**93**	**8**
Post-filter	**32**	**8**
Opportunistic sampling		
ELF	**42**	**31**
Pre-filter	**71**	**43**

Table Legend: Sample counts and unique counts are summarized by cohort and sampling method. Totals reflect the number of individual samples collected and are not normalized per subject. **Abbreviations:** epithelial lining fluid (ELF).

Table S2. CRRT Intensity and effluent flow rates in development and validation cohorts

Dataset	Ever on CRRT	Time-averaged effluent flow, L/h	Recorded effluent intensity, mL/kg/h	Observed CRRT duration, h
Development	32	2.6 (IQR 2.2-3.4; range 1.3-6)	31.2 (IQR 29.2-35.0; range 20.3-70.6)	87.9 (IQR 30.2-133.6; range 2.2-603.3)
Validation	8	2.7 (IQR 2.2-2.8; range 1.9-4)	31.5 (IQR 29.9-33.6; range 22.8-37.9)	69.6 (IQR 43.5-109.4; range 3.0-216.2)

Legend: Recorded effluent intensity is calculated as recorded absolute flow (mL/h) divided by contemporaneous weight (kg). Duration uses the stepwise CRRT indicator over observed dataset intervals.

Table S3. Forward-inclusion model comparison for cefepime population pharmacokinetic model development

Step	Structure	No. parameters	-2LL	AIC	Δ-2LL vs comparison
1	**Piecewise V1 by CRRT status; residual CL**	**10**	**1,706.804**	**1,726.804**	**—**
2	Piecewise V1; residual CL scaled by WT/70; no CrCl	10	1,710.474	1,730.474	+3.671
3	Piecewise V1; V_off scaled by WT/70; residual CL	10	1,712.845	1,732.845	+6.041
4	Piecewise V1; residual CL scaled by CrCl/120	10	1,734.984	1,754.984	+28.180

Table legend: Candidate structural models were compared using the -2 log-likelihood and AIC. The selected model retained the prespecified mechanistic covariate structure used for subsequent CRRT and body-weight simulations. **Abbreviations**: AIC, Akaike information criterion; CrCL, creatinine clearance; CRRT, continuous renal replacement therapy.

Table S4. Summary of non-parametric population PK model for cefepime in plasma and ELF

Supplemental Table. Nonparametric support points — final model

Support Point	V_{on} (L)	V_{off} (L)	V_{ELF} (L)	K_{12} (1/h)	K_{21} (1/h)	K_{15} (1/h)	K_{51} (1/h)	$CL_{systemic}$ (L/h)	S_{eff}	S_{post}	Probability	Cumulative Probability
1	7.436	8.688	44.340	4.754	7.565	3.414	1.770	1.380	0.714	0.848	0.285	0.285
2	10.519	27.431	26.726	2.906	2.762	6.806	5.377	0.948	0.906	0.738	0.178	0.463
3	15.802	9.961	23.139	5.803	9.574	2.481	9.269	1.937	0.862	1.115	0.105	0.568
4	13.043	9.435	23.248	9.754	5.815	3.664	3.895	1.399	0.638	0.576	0.093	0.661
5	7.197	8.127	42.115	4.754	7.565	1.352	1.770	1.231	0.698	0.760	0.072	0.733
6	4.771	4.987	31.458	8.040	7.043	7.881	6.499	0.697	0.816	0.876	0.068	0.801
7	5.868	4.847	38.112	6.782	6.950	8.456	5.249	0.595	0.873	1.122	0.065	0.866
8	6.315	8.104	2.858	5.689	8.096	3.789	7.583	1.241	0.876	1.152	0.043	0.909
9	29.981	17.448	98.376	7.373	9.262	0.426	9.636	1.388	1.096	0.808	0.041	0.950
10	0.697	2.906	2.877	2.472	4.909	5.352	3.895	1.701	1.182	1.148	0.034	0.985
11	23.275	21.349	45.807	1.770	5.521	8.830	6.157	2.188	0.674	0.737	0.010	0.994
12	7.436	8.688	44.301	4.754	7.565	3.414	1.770	1.380	0.714	0.848	0.002	0.996
13	29.981	17.448	99.176	7.373	9.262	0.426	9.636	1.388	1.096	0.808	0.001	0.997
14	23.275	21.349	45.846	1.770	5.521	8.830	6.157	2.188	0.674	0.737	0.001	0.997
15	4.771	4.987	31.419	8.040	7.043	7.881	6.499	0.697	0.816	0.876	0.000	0.998
16	23.275	21.349	45.787	1.770	5.521	8.830	6.157	2.188	0.674	0.737	0.000	0.998
17	10.519	27.431	26.707	2.906	2.762	6.806	5.377	0.948	0.906	0.544	0.000	0.998
18	29.981	17.448	97.654	7.373	9.262	0.426	9.636	1.388	1.096	0.808	0.000	0.998
19	7.436	8.688	44.320	4.754	7.565	3.414	1.770	1.380	0.714	0.848	0.000	0.998

Supplemental Table. Nonparametric support points — final model

Support Point	V_{on} (L)	V_{off} (L)	V_{ELF} (L)	K_{12} (1/h)	K_{21} (1/h)	K_{15} (1/h)	K_{51} (1/h)	$CL_{systemic}$ (L/h)	S_{eff}	S_{post}	Probability	Cumulative Probability
20	5.868	4.847	38.092	6.782	6.950	8.456	5.249	0.595	0.873	1.122	0.000	0.999
21	4.771	4.987	31.439	8.040	7.043	7.881	6.499	0.697	0.816	0.876	0.000	0.999
22	29.981	17.448	98.396	7.373	9.262	0.426	9.636	1.388	1.096	0.808	0.000	0.999
23	5.868	4.847	38.131	6.782	6.950	8.456	5.249	0.595	0.873	1.122	0.000	0.999
24	15.802	9.961	23.100	5.803	9.574	2.481	9.269	1.937	0.862	1.114	0.000	0.999
25	5.868	4.847	38.073	6.782	6.950	8.456	5.249	0.595	0.873	1.122	0.000	0.999
26	15.802	9.961	23.139	5.803	9.574	2.481	9.269	1.937	0.862	1.115	0.000	0.999
27	4.771	4.987	31.478	8.040	7.043	7.881	6.499	0.697	0.816	0.876	0.000	0.999
28	15.802	9.961	23.178	5.803	9.574	2.481	9.269	1.937	0.862	1.114	0.000	1.000
29	13.043	9.435	23.267	9.754	5.815	3.664	3.895	1.399	0.638	0.576	0.000	1.000
30	15.802	9.961	23.080	5.803	9.574	2.481	9.269	1.937	0.862	1.115	0.000	1.000
31	7.197	8.127	42.076	4.754	7.565	1.352	1.770	1.231	0.698	0.762	0.000	1.000
32	7.436	8.688	44.379	4.754	7.565	3.414	1.770	1.380	0.714	0.848	0.000	1.000
33	7.197	8.127	42.174	4.754	7.565	1.352	1.770	1.231	0.698	0.762	0.000	1.000
**Mean**	9.926	11.967	34.635	5.399	6.665	4.446	4.658	1.268	0.812	0.865		
**SD**	6.158	8.417	19.445	2.235	2.296	2.488	2.892	0.404	0.142	0.185		
**CV%**	62.047	70.336	56.142	41.395	34.454	55.956	62.101	31.888	17.472	21.370		

Table legend: Rows represent individual support points from the final nonparametric population model sorted by descending probability, matching the Pmetrics output. Mean, SD, and CV% summarize the final population parameter distribution. No

creatinine-clearance exponent or weight-scaled volume term is included in this final model. **Abbreviations:** V_on represents the CRRT-on central volume term; V_off represents the CRRT-off central volume term; V_ELF represents the ELF scaling volume; CL_systemic represents native systemic clearance; S_eff represents the CRRT effluent clearance scalar; S_post represents the post-filter plasma scalar. CrI: credible interval; CV%: coefficient of variation as percentage.

Figure S1. Structural cefepime population pharmacokinetic model for plasma, ELF, and CRRT compartments

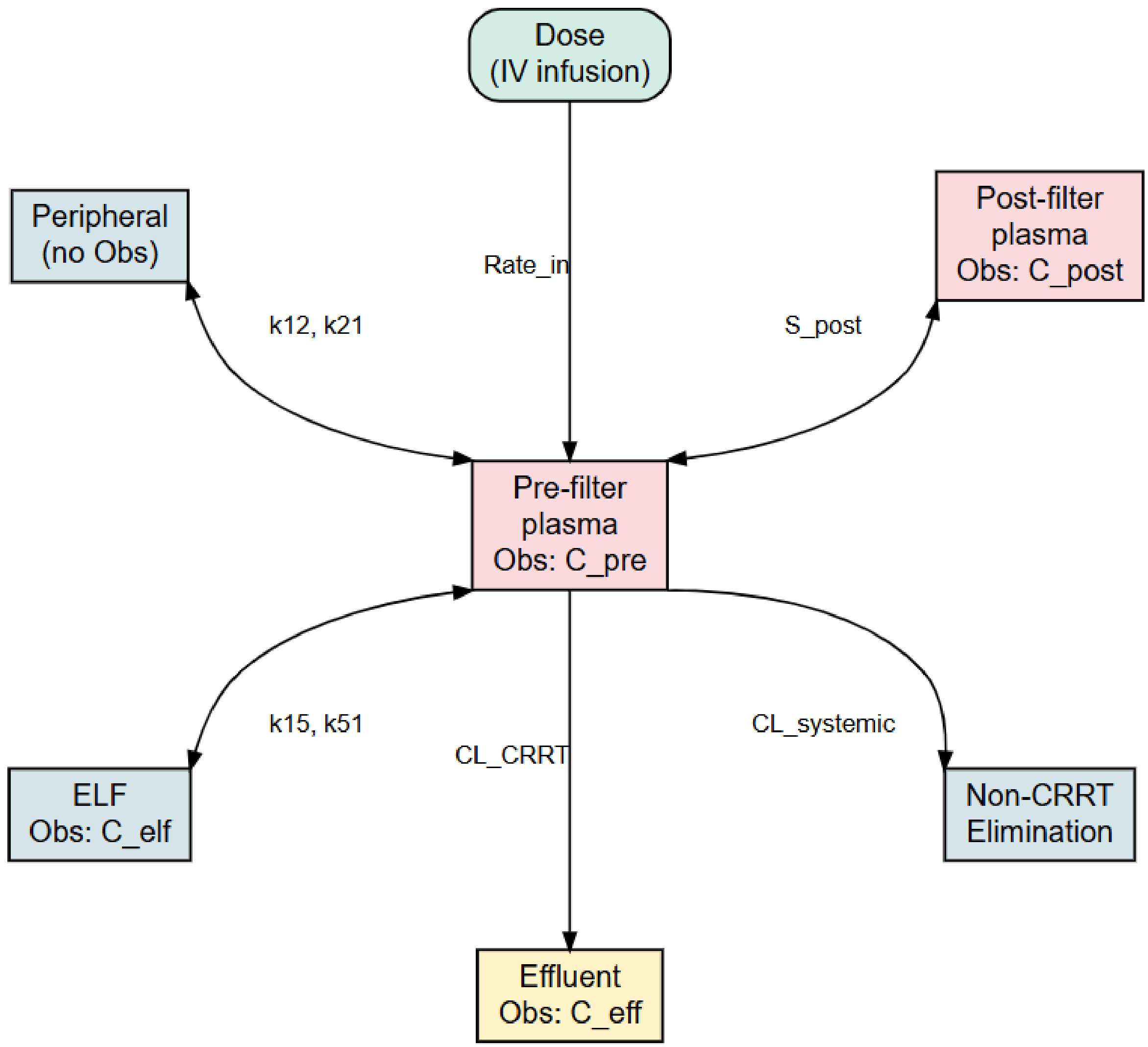

Figure legend: Schematic of cefepime population PK model showing intravenous input into the pre-filter plasma compartment, distribution to the peripheral and ELF compartments, post-filter plasma scaling, and elimination through systemic and CRRT effluent pathways. Abbreviations: CRRT, continuous renal replacement therapy; ELF, epithelial lining fluid; IV, intravenous; Obs, observed concentration.

Figure S2. Posterior weighted prediction error diagnostics in the validation cohort

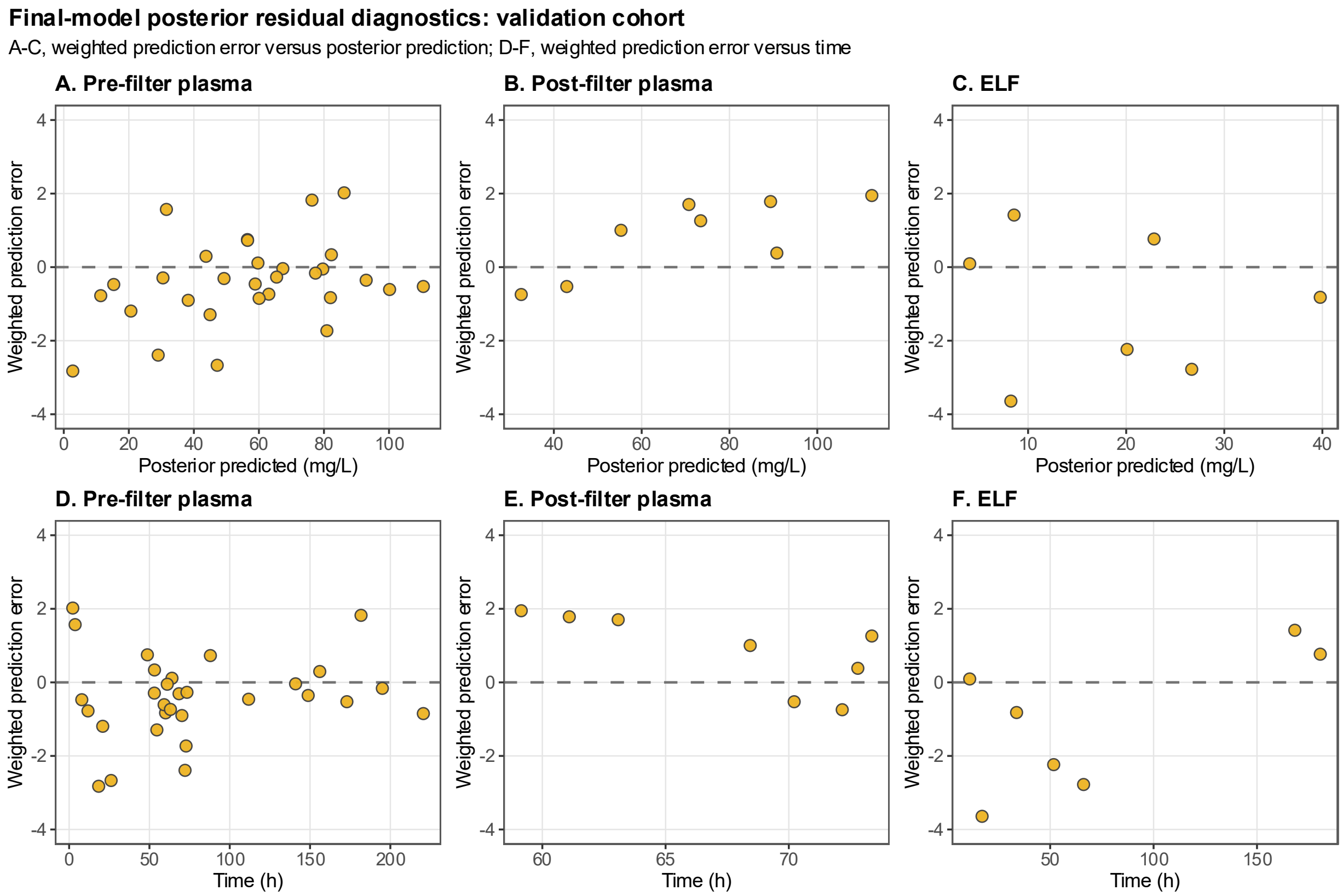

Figure legend: Weighted prediction errors are shown versus posterior individual predictions for pre-filter plasma (A), post-filter plasma (B), and epithelial lining fluid (C), and versus time for pre-filter plasma (D), post-filter plasma (E), and epithelial lining fluid (F). Posterior predictions were based on the final population pharmacokinetic model using median individual parameter estimates. Dashed horizontal lines indicate a weighted prediction error of zero.

Figure S3. Cefepime PTA by MIC for 2g q12h intermittent infusion with loading dose

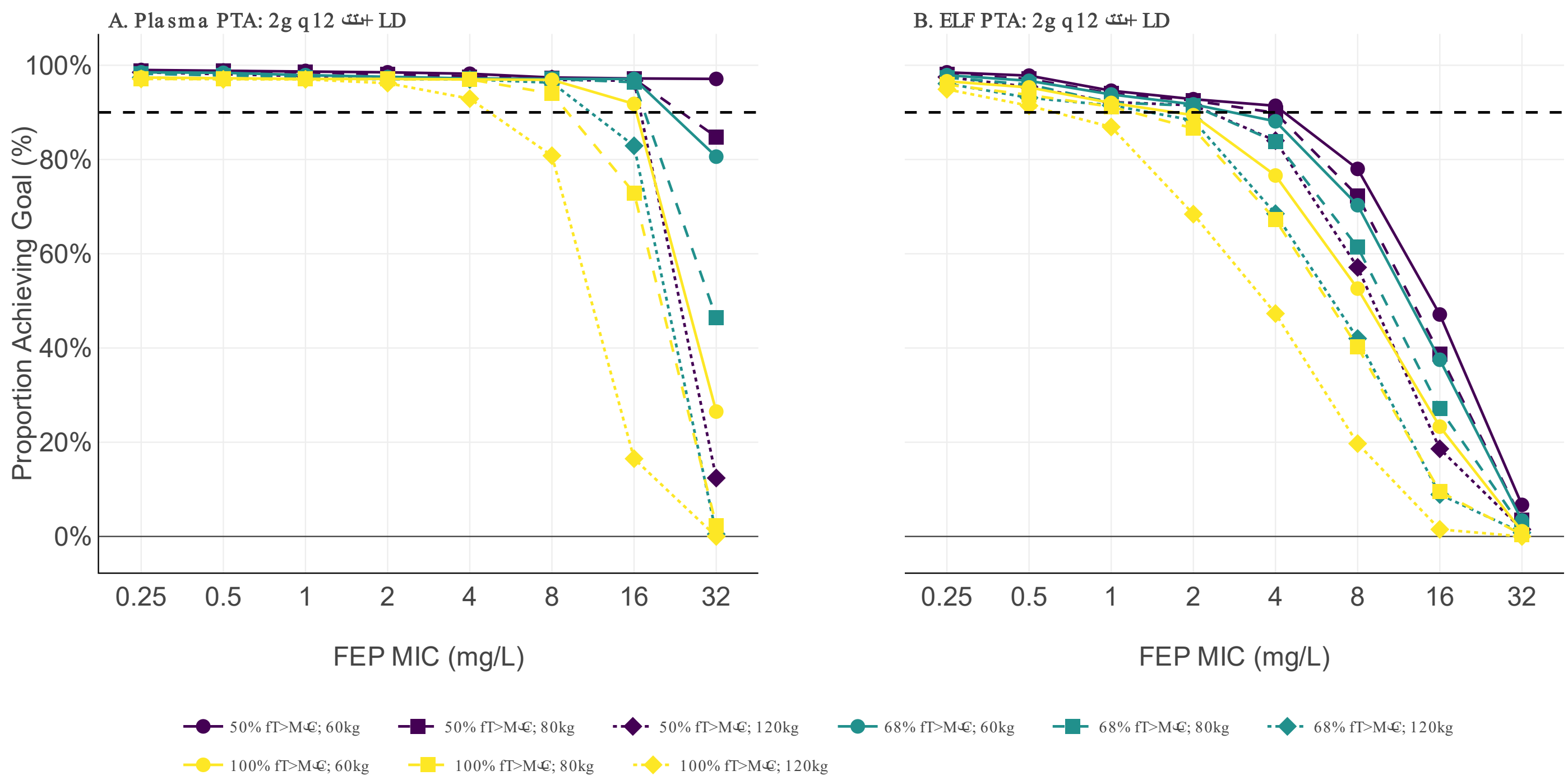

Figure legend: Probability of target attainment across cefepime MICs for plasma (A) and epithelial lining fluid (B) after 2g q12h intermittent infusion (II) with a loading dose. Lines are stratified by body weight and pharmacodynamic target. The dashed horizontal line indicates 90% PTA. Abbreviations: II, intermittent infusion; ELF, epithelial lining fluid; FEP, cefepime; LD, loading dose; MIC, minimum inhibitory concentration; PTA, probability of target attainment. Clinical susceptibility breakpoints for cefepime vary by organism and interpretive standard; therefore, no single susceptibility breakpoint is shown for these organism-independent PTA simulations.

Figure S4. Cefepime PTA by MIC for 2 g q12h extended infusion with loading dose

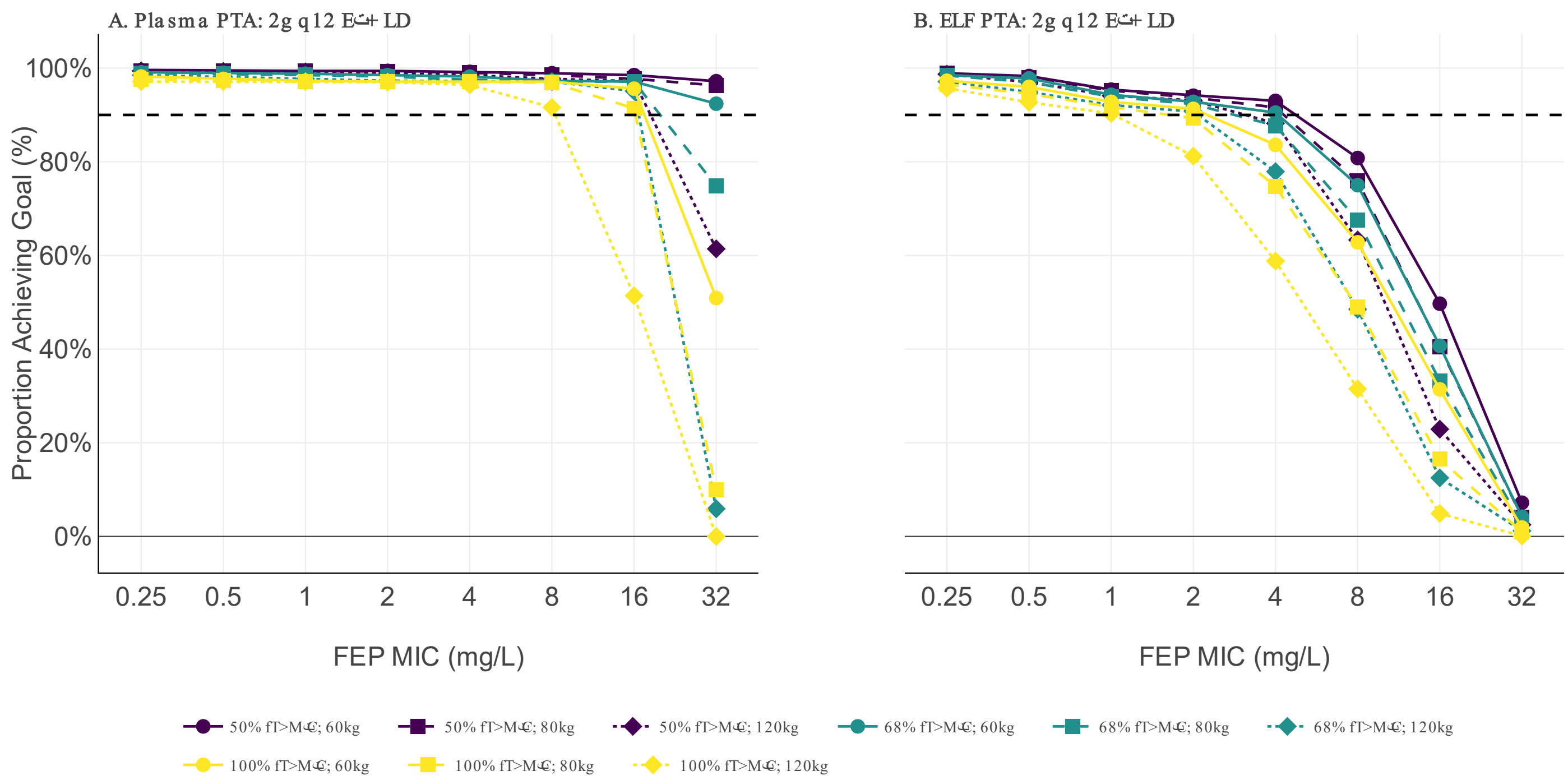

Figure legend: Probability of target attainment across cefepime MICs for plasma (A) and epithelial lining fluid (B) after 2g q12h extended infusion (EI) with loading dose. Lines are stratified by body weight and pharmacodynamic target. The dashed horizontal line indicates the 90% PTA benchmark. **Abbreviations**: EI, extended infusion; ELF, epithelial lining fluid; FEP, cefepime; LD, loading dose; MIC, minimum inhibitory concentration; PTA, probability of target attainment. Clinical susceptibility breakpoints for cefepime vary by organism and interpretive standard; therefore, no single susceptibility breakpoint is shown for these organism-independent PTA simulations.

Figure S5. Cefepime PTA by MIC for 4g q24h continuous infusion with loading dose

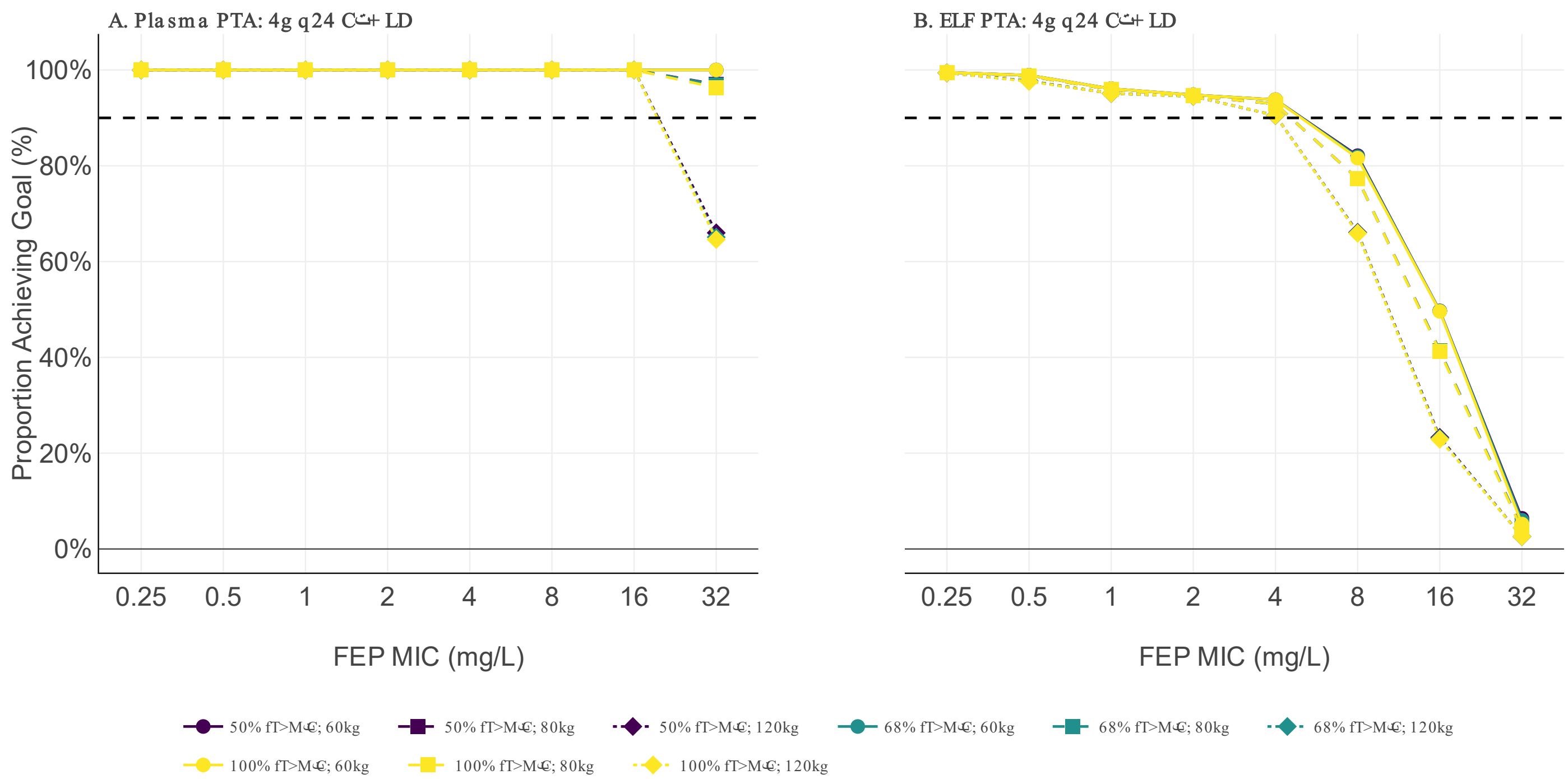

Figure legend: Probability of target attainment across cefepime MICs for plasma (A) and epithelial lining fluid (B) after 4g q24h continuous infusion (CI) with a loading dose. Lines are stratified by body weight and pharmacodynamic target. The dashed horizontal line indicates the 90% PTA benchmark. **Abbreviations**: CI, continuous infusion; ELF, epithelial lining fluid; FEP, cefepime; LD, loading dose; MIC, minimum inhibitory concentration; PTA, probability of target attainment. Clinical susceptibility breakpoints for cefepime vary by organism and interpretive standard; therefore, no single susceptibility breakpoint is shown for these organism-independent PTA simulations.
